# Reconstructing each cell's genome within complex microbial communities—dream or reality?

**DOI:** 10.3389/fmicb.2014.00771

**Published:** 2015-01-08

**Authors:** Scott Clingenpeel, Alicia Clum, Patrick Schwientek, Christian Rinke, Tanja Woyke

**Affiliations:** DOE Joint Genome InstituteWalnut Creek, CA, USA

**Keywords:** multiple displacement amplification, single-cell sequencing, genome completeness, genome quality, microbial ecology, environmental microbiology

## Abstract

As the vast majority of microorganisms have yet to be cultivated in a laboratory setting, access to their genetic makeup has largely been limited to cultivation-independent methods. These methods, namely metagenomics and more recently single-cell genomics, have become cornerstones for microbial ecology and environmental microbiology. One ultimate goal is the recovery of genome sequences from each cell within an environment to move toward a better understanding of community metabolic potential and to provide substrate for experimental work. As single-cell sequencing has the ability to decipher all sequence information contained in an individual cell, this method holds great promise in tackling such challenge. Methodological limitations and inherent biases however do exist, which will be discussed here based on environmental and benchmark data, to assess how far we are from reaching this goal.

## Introduction

Our current inability to cultivate the bulk of bacteria and archaea in the laboratory combined with a strong drive to analyze microbial communities *in situ*, gave rise to a large array of cultivation-independent methods that have been used for the past 20 years. These have been crucial to study microbial communities by deciphering community structure, organization and function. Commonly used techniques include microarrays, fluorescence *in situ* hybridization (FISH) and PCR-based approaches. With the advent of metagenomics it became possible to examine the genetic content of microbial communities without needing any *a priori* knowledge of the genetic sequences present. Although improved methods for the assembly and binning of metagenome sequences are emerging (Wrighton et al., 2012; Albertsen et al., 2013), linking potential functions to phylogeny still poses a major challenge for metagenomic approaches, especially in complex communities. One way to simplify such complex systems is to focus on the single microbial cell, which is the basic structural and functional unit of living organisms. Single-cell genomics is a method that allows the linkage of function to phylogeny while avoiding the difficulties in cultivating microorganisms.

An array of review articles exists describing the state of the art single-cell microbial genomics (de Jager and Siezen, 2011; Lasken, 2012; Stepanauskas, 2012; Yilmaz and Singh, 2012; Blainey, 2013; Lasken and McLean, 2014), outlining key challenges, proposing potential solutions and summarizing the accomplishments that have been achieved with this technology. Using several environmental samples as well as reference organisms for benchmarking, we here place single-cell genomics in perspective and discuss how far we are from reconstructing each individual cell's genome within an environmental sample. We provide some practical implications of using the technology by exposing some of the current biases and limitations while bearing in mind the tremendous window of opportunity.

## Limited genome access

**Figure 1:**
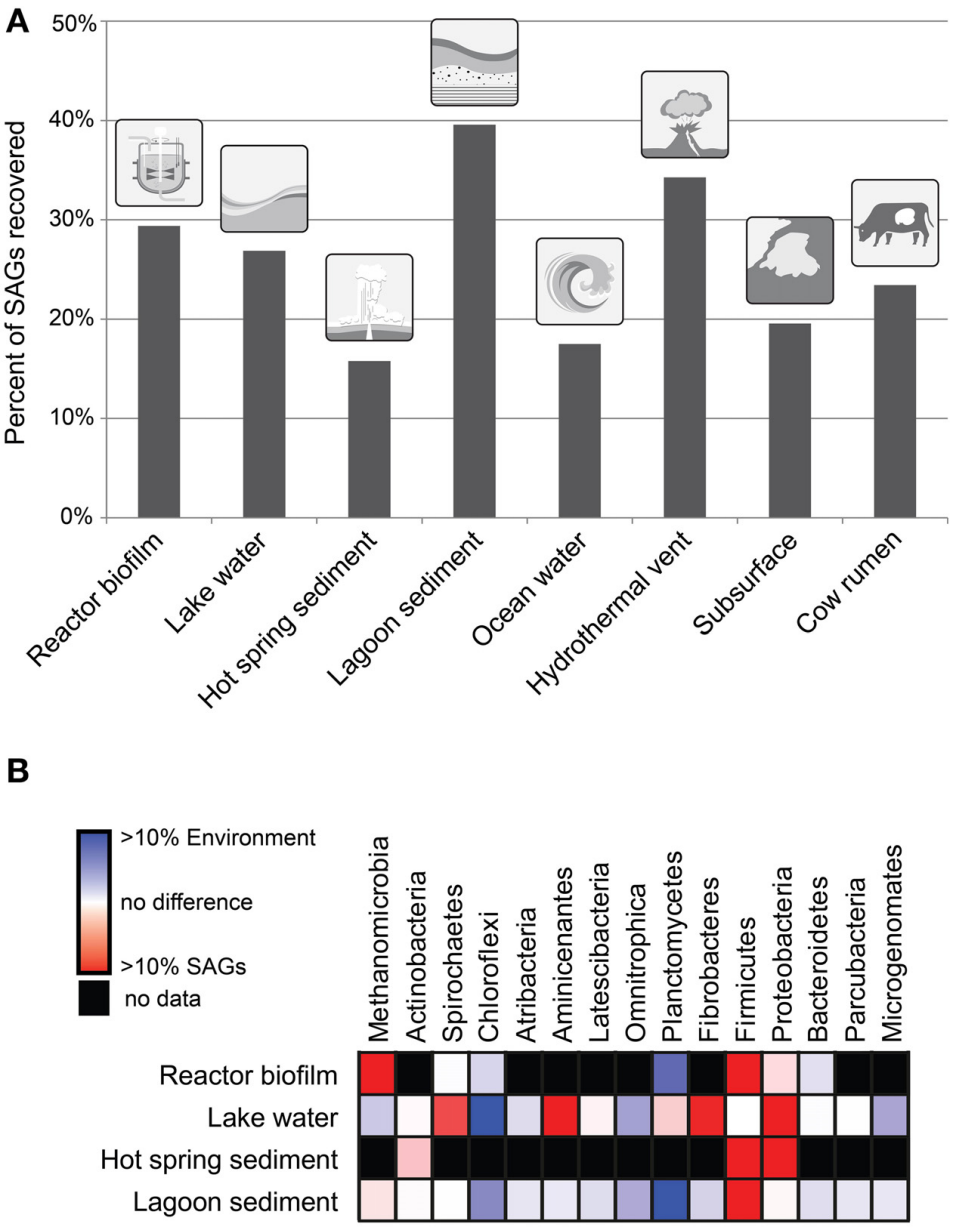
**(A)** Percentage of single cells sorted from a variety of environmental samples that were successfully amplified by MDA. A minimum of 800 single cells were sorted for each of these sites. **(B)** Abundance of taxa in 16S rRNA gene tag sequence data as compared to the SAG libraries for four diverse environmental samples. Taxa represent phyla of bacteria and the archaeal class Methanomicrobia.

The fraction of single-cell genomes that can be recovered from a sample is highly variable (Figure 1A) due to technical challenges and biases at multiple steps of the process. The first step involves sample preparation and the isolation of single cells. Each sample may need custom sample preparation methods depending on the nature of the sample. While generalized recommendations do exist (Rinke et al., 2014), research should be done on which methods for dispersing the cells works best with differing sample types. High throughput single-cell isolation is generally performed using fluorescence activated cell sorting (FACS). This typically involves sorting based on the size of the particle (determined by a scatter signal) and fluorescence of a DNA stain such as SYBR Green. In principle every cell from a sample could be sorted, but practical limitations come from difficulties in dispersing the cells (cells attached to particles, aggregated cells), the inability to sufficiently stain all types of cells, and cells that fall outside the sorting window due to odd shapes (e.g., filaments) or unusually large or small size.

The next step of the process involves lysing the cells so that the genome amplification reagents will have access to the cell's DNA. High variability in the composition of microbial cell walls makes it difficult to find a universal method for cell lysis. This is further complicated by the nature of working with single cells. Since there may only be one copy of the genome, any nicks or double-stranded breaks in the DNA prior to genome amplification will likely lead to gaps in the resulting assembly. It therefore is necessary to use lysis methods that are gentle enough to maintain the integrity of the DNA. Moreover, clean-up steps are impractical due to the small sample and reaction size. Thus, reagents added to the cell for lysis will remain there during the genome amplification step. The lysis reagents therefore have to be compatible with the multiple displacement amplification (MDA) chemistry. Even if a cell is successfully lysed it is possible that the phi29 polymerase will not have access to copy the DNA, possibly due to supercoiling of the DNA or nucleoid-associated proteins or other proteins being bound to the DNA. Research is needed on the magnitude of impact that proteins bound to the DNA such as nucleoid-associated proteins have on blocking the phi29 polymerase. Perhaps an additional step of protease treatment will significantly improve the recovery of genomes from some groups of microbes.

Currently, the most commonly used single-cell lysis method involves an alkaline treatment, which some taxa are not susceptible to, leaving great potential for improvement in this step of the process. Physical methods for lysing cells such as sonication or freeze-thaw cycles are by their nature the most universal ways to lyse cells. However, they are also the methods most likely to shear the DNA. Chemical lysis methods can be less damaging to DNA, but are more sensitive to the composition of the cell wall. Finally, enzymatic methods to degrade the cell wall are the least likely to damage the DNA, but they are the most specific to cell wall composition. A cocktail of enzymes could be used to increase the diversity of cells lysed. One challenge with using enzymes is that they are manufactured by living organisms, which almost inevitably introduces contaminant DNA from the production organism. Thorough quality control and clean-up is thus recommended prior to use for single-cell lysis.

## Biases in the diversity

The current process of cell sorting involves an anonymous sort meaning that any particle that is within the correct size range and is sufficiently stained is sorted. This results in a random selection of single cells from the sample. Targeted sorting of particular groups of cells is rarely done because it requires a sufficiently bright stain or label. Many stains that work well with microscopy where exposure times can be relatively long are not suitable for FACS since the cell is only in the detection window for less than 100 μs. Thus, only after the genome amplification is completed is there sufficient material to perform screening steps to identify the single amplified genomes (SAGs) obtained; usually by amplification and sequencing of the 16S rRNA gene. Some single-cell MDA products will not yield 16S rRNA gene PCR amplification products. Reasons for this can include the lack of these genomics regions in the amplified DNA due to the amplification bias (see below) or mismatches of the universal 16S rRNA primers, which will prevent amplification of 16S rRNA genes in certain taxa (e.g., Baker et al., 2003, 2010; Youssef et al., 2014).

The 16S rRNA gene identification step could potentially be improved by optimizing the PCR reaction conditions. Multiple primer sets could be used to reduce the bias of any one particular primer set. PCR and sequencing of additional marker genes could be added to account for cases where the MDA amplification bias causes a lack of the 16S rRNA gene. However, these steps would increase the cost and amount of work for each single-cell genome that was amplified and may require separate optimization for each sample. Perhaps a more efficient way to deal with the biases introduced by the 16S rRNA gene identification step is to skip it entirely. The continual decrease in library generation and sequencing costs is making it more feasible to sequence all MDA products without requiring identification by PCR and sequencing of the 16S rRNA gene. Improved methods for targeted sorting of particular groups of interest would also allow one to skip the biases inherent to the 16S rRNA gene identification step since every sorted cell would represent a cell of interest.

All of the difficulties and biases given above in conjunction with 16S rRNA gene PCR biases, lead to a discrepancy in the diversity recovered by single-cell genomics compared to that seen in 16S rRNA gene tag data (Figure 1B). Based on four environmental samples analyzed, we found some groups such as the Proteobacteria are consistently overrepresented in the SAG library (see Supplementary Material for methods). These taxa appear to be quite amenable to single-cell genomics by being generally easy to sort and lyse. Other groups such as the Chloroflexi are consistently underrepresented possibly due to their often filamentous morphology, high GC contents, or having unusually tough cell walls. As more data from additional environments is collected in the future, these biases and their underlying cause will likely become clearer.

## Genome recovery

In addition to the limitations on the fraction of cells for which one can generate single-cell genomes, there is a current constraint on quality of the genome that can be recovered by single-cell sequencing. To explore this we produced single-cell genomes from three strains of bacteria with differing genomic G+C contents: *Pedobacter heparinus* DSM 2366, 42% GC; *Escherichia coli* K12-MG1655, 51% GC; and *Meiothermus ruber* DSM 1279, 63% GC (Clingenpeel et al., 2014). These have complete genome sequences available and have the same cell wall structure (Gram negative) to control for major differences in lysis efficiency. For each strain, we sequenced 8 single cells.

**Figure 2:**
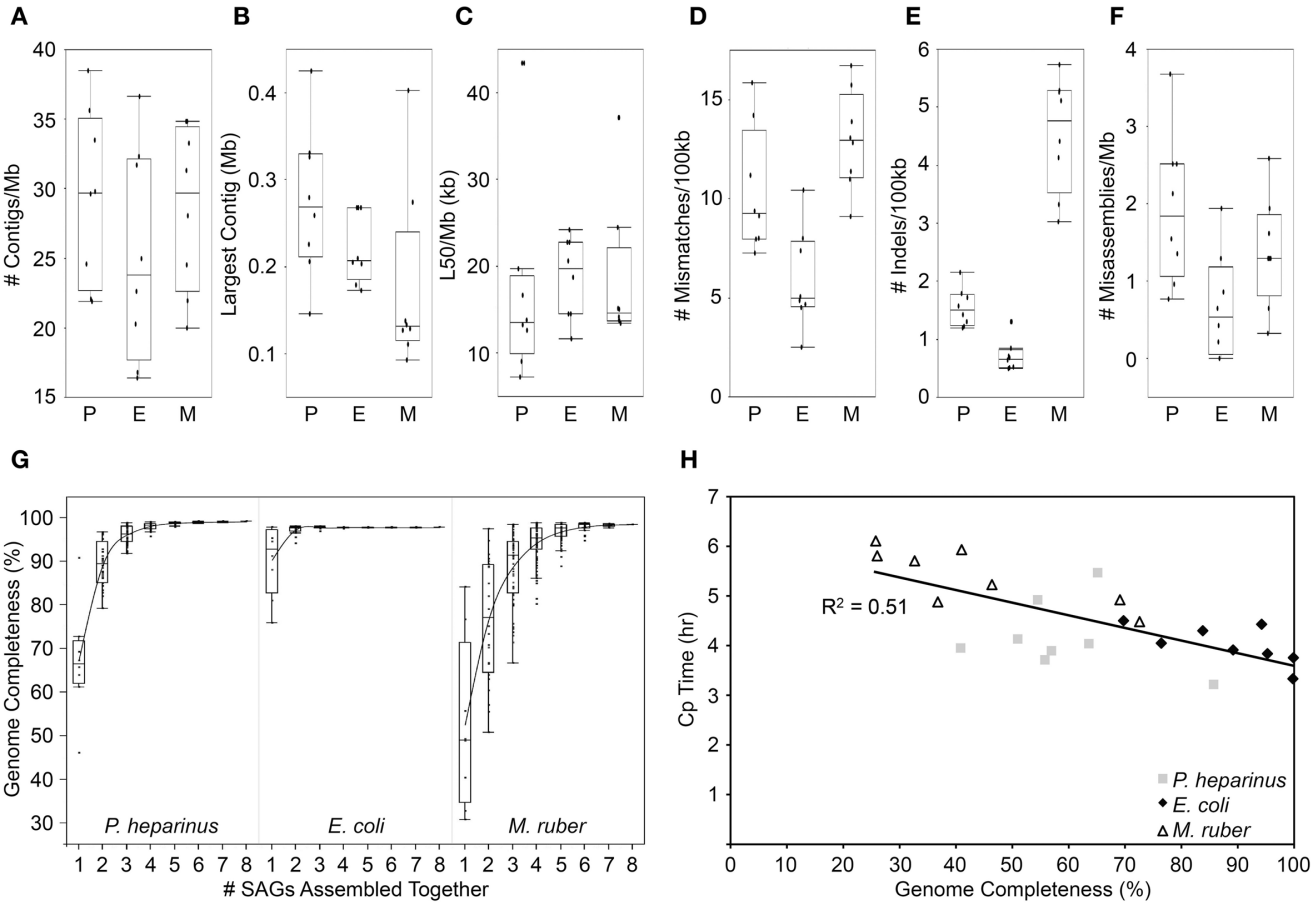
**Benchmark single-cell experiment using three reference strains with finished genomes: P, *Pedobacter heparinus;* E, *Escherichia coli;* and M, *Meiothermus ruber***. **(A)** Number of assembled contigs >2 kb in length normalized by genome size. **(B)** Largest assembled contig. **(C)** The length of the shortest contig among those that collectively cover half of the assembly (L50) normalized by genome size. **(D)** Number of mismatches between the assembly and the reference genome normalized by genome size. **(E)** Number of insertions and deletions in the assembly when compared to the reference genome normalized by genome size. **(F)** Number of misassemblies when compared to the reference genomes normalized by genome size. **(G)** Percentage of the genome recovered in the assembly when the reads from multiple SAGs are combined together and assembled. **(H)** Genome recovery vs. real-time MDA crossing point (CP) value.

The median amount of the genome recovered was 66% for *P. heparinus*, 93% for *E. coli*, and 49% for *M. ruber*. All three strains had comparable numbers and sizes of contigs in their assemblies when normalized to their genome size (Figures 2A–C). It is possible that the lower genome recovery in *M. ruber* is due to its higher GC content. Other possible reasons include partial lysis or limited genome access due to DNA-bound proteins. Since *M. ruber* is somewhat more alkaliphilic than the other two strains (optimal growth at pH 8; Nobre et al., 1996) the alkaline treatment used to lyse the cells and denature the contents may have been less effective than in the other two strains. The MDA reaction itself can provide an indication of the completeness of the genome to aid in selecting which SAGs should be fully shotgun sequenced. If the MDA reactions are monitored in real time (similar to RT-PCR) then the time at which the inflection point of the real-time amplification curves occurs (crossing point; CP value) is correlated with the completeness of the genome obtained (Figure 2H).

The amount and quality of a single-cell genome recovered is also partly dependent on how the sequencing reads are assembled. The nature of single-cell sequence is a result of unique complications largely introduced during the whole genome amplification. The MDA process results in uneven coverage across the genome. Parts of the genome can have tens of thousands-fold coverage while other regions have less than ten-fold coverage. Traditional sequence assemblers rely on even coverage across the genome and thus perform poorly on SAG datasets. One way of dealing with this is to normalize the coverage by removing excess sequences from the high coverage regions either in the lab or *in silico* (Rodrigue et al., 2009; Swan et al., 2011; Rinke et al., 2013). More recently, assemblers have been developed specifically to handle the variable sequence coverage including SPAdes, EULER+Velvet-SC and IDBA-UD (Chitsaz et al., 2011; Bankevich et al., 2012; Peng et al., 2012). When various assemblers are tested on SAG data from organisms with complete reference genomes SPAdes tends to produce the largest assemblies with the lowest error rates (Nurk et al., 2013). Using SPAdes 3.1.0 to assemble our benchmark SAG datasets, the pattern of errors follows that of genome completeness. *E. coli* had the most complete genomes and the lowest error rates while *M. ruber* had the least complete genomes and the highest error rates (Figures 2D–G). When compared to 25 isolate genomes generated at the JGI (data not shown) the *E. coli* error rates are not significantly different from the isolates, but both the *P. heparinus* and *M. ruber* are significantly higher (*P* < 0.01). For those two strains the average number of mismatches per 100 kb is 3–4x higher than the isolates, the average number of indels per 100 kb is 3–9x higher than in the isolates and the average number of misassemblies is 4–5x higher than in the isolates.

The recovery of partial genomes from single cells is due to parts of the genome being significantly over amplified compared to other regions. This is believed to be random process (Marcy et al., 2007; Lasken, 2012; Yilmaz and Singh, 2012; Lasken and McLean, 2014). If the bias is indeed completely random, different single cells from the same strain should recover different portions of the overall genome, thus complementing each other when being co-assembled. We co-assembled all eight SAGs for each strain using SPAdes 3.1.0. The more complete the individual SAGs were, the fewer SAGs were required to be combined to produce a near complete genome. A median completeness of >97% was obtained when 2 *E. coli*, 4 *P. heparinus*, and 5 *M. ruber* SAGs were combined (Figure 2G).

While the data described above demonstrate that combined assemblies are feasible with axenic cultures allowing the recovery of more complete genomes, the question of whether they are advantageous for environmental samples remains. Combined assemblies have been performed with single cell genomes from environmental samples (Blainey et al., 2011; Dodsworth et al., 2013; Rinke et al., 2013). The general selection criterion for co-assembly of environmental SAGs has been an average nucleotide identity of ≥95%, which is being used to delineate species (Konstantinidis and Tiedje, 2005; Goris et al., 2007; Richter and Rossello-Mora, 2009). However, one has to bear in mind that the resulting consensus assemblies are composite genomes representing a population and not a single organism, in which single nucleotide polymorphisms (SNPs) may become collapsed in the resulting consensus sequence.

Instead of dealing with the issue of partial genome recovery after the fact by combining multiple sequenced single-cell genomes, it is desirable to try to maximize the amount of genome recovered in the first place by minimizing the amplification bias inherent to MDA. One way to mitigate the amplification bias is to start with multiple copies of the target genome. Two approaches to do this have been successfully demonstrated. The first is to artificially increase the number of copies of the genome in a cell. Such artificial polyploidy involves inhibiting the FtsZ protein which is critical for cell division, while allowing genome replication to continue (Dichosa et al., 2012). This method has the potential to be broadly applicable since FtsZ is found in most bacteria and euryarchaea that have been examined. However, this protein has not been found in other archaeal groups and it may be absent in uncultivated phyla, which are the taxonomic groups for which single-cell sequencing is most useful. As each FtsZ inhibitor discovered so far has its own range of taxa that it will affect, it is also unclear whether a cocktail of inhibitors can be found that will work with the full diversity of known FtsZ proteins. The growth of cells into microcolonies represents another method to improve the number of copies of the genome (Zengler et al., 2002; Fitzsimons et al., 2013; Dichosa et al., 2014). This process involves isolating individual cells in gel microdroplets (GMDs) and then allowing them to replicate in a co-culture with other cells from the environment while still maintaining a clonal population within each GMD. Even a few cell doublings would result in enough copies of the genome to substantially reduce the amplification bias. The process of co-culturing allows the cells to exchange metabolites and chemical signals with other cells, which has the potential to produce growth in a larger fraction of organisms as compared to traditional cultivation methods.

Other approaches to minimize amplification bias target the amplification reaction itself. Several strategies have been attempted to reduce the amplification bias including shrinking reaction volumes, adding molecular crowding agents, or changing the amplification method itself. Reducing reaction volumes by using a microfluidics system (Marcy et al., 2007) or microwells (Gole et al., 2013) have been reported to reduce the bias. In addition to reducing amplification bias, shrinking the reaction volume limits the risk of reagent-based DNA contamination. Although the use of microfluidics systems allows a significant reduction in reaction volume, such systems are currently not high throughput. Instruments such as the Labcyte Echo liquid handling system permit the accurate transfer of sub-microliter volumes which allows the shrinking of volume of the MDA reaction while maintaining a high throughput (Rinke et al., 2014). The addition of trehalose (Pan et al., 2008) or polyethylene glycol 400 (Ballantyne et al., 2006) to the MDA reaction improves the amount and evenness of the genome recovered. These are thought to work as molecular crowding agents, which increase the initial binding of primers and polymerase throughout the template. This would reduce the stochastic over-amplification of a few regions that started amplifying first in an uncrowded reaction. Although the phi29 polymerase used in MDA has numerous advantages, there is the possibility that other amplification chemistries could prove to be superior. One alternative is multiple annealing and looping-based amplification cycles (MALBAC; Zong et al., 2012). This method reduces the bias from exponential amplification seen in MDA by using a quasilinear preamplification step. While the MALBAC method had favorable results with a human cancer cell line (Zong et al., 2012), it did not perform well compared to MDA when applied to single *E. coli* cells (de Bourcy et al., 2014). While limiting the MDA by reducing the volume of the reaction or the time of the reaction reduces bias, it results in lower amounts of DNA available for sequencing. Improvements in library creation methods such as the Illumina Nextera XT sample preparation kit can create sequencing libraries from sub-nanogram amounts of input DNA, which makes limited MDA a viable approach to reducing amplification bias.

## Outlook

From a genomics perspective, the recovery of complete genome sequences from single cells and from each cell within a complex environment is a desirable goal. Yet current technical difficulties and limitations still prevent this. New innovations will inevitably emerge, which may range from new, improved enzymes and chemistries to amplify a single-cell genome to novel single molecule sequencing technologies that may eliminate the need of sequencing library creation, thus removing the need for whole genome amplification entirely. For the time being, we will have to settle with partial genomes from a fraction of the cells contained within an environmental sample and reconstructing each cell's genome from a complex environment still remains a dream rather than reality. However, these partial single-cell genomes are clearly pushing microbial genomics into exciting, untapped territory, enabling the discovery of unexpected metabolic features, providing insights into population genetics, and improving the phylogeny of microbes (e.g., Swan et al., 2011; Rinke et al., 2013; Kashtan et al., 2014; Roux et al., 2014). We expect important discoveries will continue to be made as single-cell sequencing is applied to more environments.

## Author contributions

Tanja Woyke conceived and managed the project. Scott Clingenpeel and Christian Rinke performed the experiments. Alicia Clum and Patrick Schwientek analyzed the data. Scott Clingenpeel, Alicia Clum, Patrick Schwientek, Christian Rinke, and Tanja Woyke wrote the paper.

### Conflict of interest statement

The authors declare that the research was conducted in the absence of any commercial or financial relationships that could be construed as a potential conflict of interest.
