# Supplementary material for: Reconstructing each cell's genome within complex microbial communities—dream or reality?
Source: Front Microbiol. 2015 Jan 8;5:771. doi: 10.3389/fmicb.2014.00771 (PMC4287102; doi:10.3389/fmicb.2014.00771)
Supplement: Supplementary file 1 [file DataSheet1.DOCX]

**Supplementary material**

**Methods**

Single-cell genomes and 16S rRNA gene tag sequences for Figure 1 were obtained as described in Rinke et al. (2013). Briefly, separate aliquots of each sample were used to produce single amplified genomes (SAGs) and 16S rRNA gene pyrotag sequences. The pyrotag sequences were generated on a Roche 454 FLX-titanium sequencer using primers 926F/1392R which target the V6-V8 region of the 16S rRNA gene. The same primer set was used to identify the SAGs. Both the pyrotag and SAG 16S rRNA gene sequences were taxonomically classified to the phylum level for bacteria and to the class level for archaea.

For Figure 2, single-cell genomes were obtained as described in Clingenpeel et al. (2014). Reads were subsampled to 315x per genome. Reads were filtered for human contamination by alignment using bwa (Li and Durbin, 2010) and for Illumina artifacts using an in-house tool duk (unpublished). The remaining reads were assembled with SPAdes version 3.1.0 with --sc –careful (Bankevich et al., 2012). Contigs smaller than 2,000 bp were removed. Assembly statistics were generated using QUAST version 2.3 build 28.02.2014 (Gurevich et al., 2013).

**References**

Bankevich, A., Nurk, S., Antipov, D., Gurevich, A. A., Dvorkin, M., Kulikov, A. S., et al. (2012). SPAdes: a new genome assembly algorithm and its applications to single-cell sequencing. *J. Comput. Biol.* 19, 455-477. doi: 10.1089/cmb.2012.0021

Clingenpeel, S., Schwientek, P., Hugenholtz, P., and Woyke, T. (2014). Effects of sample treatments on genome recovery via single-cell genomics. *ISME J.* available online ahead of print. doi: 10.1038/ismej.2014.92

Gurevich, A., Saveliev, V., Vyahhi, N., and Tesler, G. (2013). QUAST: quality assessment tool for genome assemblies. *Bioinformatics* 29, 1072-1075. doi: 10.1093/bioinformatics/btt086

Li, H., and Durbin, R. (2010). Fast and accurate long-read alignment with Burrows-Wheeler transform. *Bioinformatics* 26, 589-595. doi: 10.1093/bioinformatics/btp698

Rinke, C., Schwientek, P. Sczyrba, A., Ivanova, N. N., Anderson, I. J., Cheng, J.-F. et al. (2013). Insights into the phylogeny and coding potential of microbial dark matter. *Nature* 499, 431-437. doi: 10.1038/nature12352
